# Identification of a de novo splicing variant in the Coffin–Siris gene, *SMARCE1,* in a patient with Angelman‐like syndrome

**DOI:** 10.1002/mgg3.511

**Published:** 2018-12-11

**Authors:** Cinthia Aguilera, Elisabeth Gabau, Steve Laurie, Neus Baena, Sophia Derdak, Núria Capdevila, Ariadna Ramirez, Veronica Delgadillo, Maria Jesus García‐Catalan, Carme Brun, Miriam Guitart, Anna Ruiz

**Affiliations:** ^1^ Genetics Laboratory UDIAT‐Centre Diagnòstic Parc Taulí Hospital Universitari Institut d'Investigació i Innovació Parc Taulí I3PT Universitat Autònoma de Barcelona Sabadell Spain; ^2^ Paediatric Unit Parc Taulí Hospital Universitari Institut d'Investigació i Innovació Parc Taulí I3PT Universitat Autònoma de Barcelona Sabadell Spain; ^3^ CNAG‐CRG Centre for Genomic Regulation (CRG) The Barcelona Institute of Science and Technology Barcelona Spain

**Keywords:** Angelman syndrome (AS), Coffin–Siris syndrome (CSS), exome sequencing, *SMARCE1*

## Abstract

**Background:**

Patients affected by Angelman syndrome (AS) present severe intellectual disability, lack of speech, ataxia, seizures, abnormal electroencephalography (EEG), and a characteristic behavioral phenotype. Around 10% of patients with a clinical diagnosis of AS (AS‐like) do not have an identifiable molecular defect. Some of these patients harbor alternative genetic defects that present overlapping features with AS.

**Methods:**

Trio whole‐exome sequence was performed on patient and parent's DNA extracted from peripheral blood. Exome data were filtered according to a *de novo* autosomal dominant inheritance. cDNA analysis was carried out to assess the effect of the splice site variant.

**Results:**

We identified a novel heterozygous *SMARCE1* splicing variant that leads to an exon skipping in a patient with an Angelman‐like phenotype. Missense variants in the *SMARCE1* gene are known to cause Coffin–Siris syndrome (CSS), which is a rare congenital syndrome. Clinical reevaluation of the patient confirmed the presence of characteristic clinical features of CSS, many of them overlapping with AS.

**Conclusions:**

Taking into account the novel finding reported in this study, we consider that CSS should be added to the expanding list of differential diagnoses for AS.

## 1 INTRODUCTION

Angelman syndrome (AS) is a neurodevelopmental disorder characterized by severe intellectual disability, lack of speech, ataxia of gait, seizures, a characteristic electroencephalography (EEG), and a unique behavior that includes any combination of happy demeanor, easily excitable personality, frequent laughter, and stereotypes (Bird, 2014; Buiting, 2010). AS is caused by the lack of expression of the maternally inherited *UBE3A* gene (OMIM 601623) in neurons. Around 10% of patients with a clinical diagnosis of AS are not molecularly confirmed (Dagli, Buiting, & Williams, 2012). Some of these Angelman‐like syndrome patients harbor alternative genetic defects that present overlapping clinical features with AS (Tan, Bird, Thibert, & Williams, 2014).

Genomic approaches such as array comparative genomic hybridization and whole‐exome sequencing have already been useful to identify alternative genes responsible for other heterogeneous genetic diseases such as Rett, Kleefstra, and Smith–Magenis syndromes (Berger et al., 2017; Kleefstra et al., 2012; Sajan et al., 2016). Here, we identified with exome sequencing a novel heterozygous *SMARCE1* (OMIM 603111) splicing variant in a patient with an Angelman‐like phenotype.

## 2 MATERIALS AND METHODS

### 2.1 Ethical compliance

The protocol for the study has been approved by the institutional Ethics Committee of Institut d'Investigació i Innovació Parc Taulí I3PT and the corresponding informed consent has been obtained from the parents.

### 2.2 Patient

The proband is a 14‐year‐old boy who was born at term to non‐consanguineous parents following a normal pregnancy. The patient had sucking difficulties during the neonatal period. At the age of 3 months, he suffered from seizures and at 6 months, he was found to be hypotonic. He presented global developmental delay: He sat unsupported at 12 months, walked independently at 27 months, and speaks only three words. Dentition was delayed. At 2 years old, neurological examination detected severe intellectual disability, ataxia of gait, receptive and non‐verbal communication skills higher than verbal ones and frequent drooling. He presented an abnormal electroencephalogram (EEG), although it was not the characteristic found in AS patients. The behavioral phenotype included happy demeanor, easily excitable personality, hyperactivity, attention deficit, stereotypies, attraction to water, aggressiveness, and autistic features. A clinical suspicion of AS was raised which was not confirmed molecularly. AS testing included methylation PCR of the 15q11.2‐q13 region, *UBE3A* sequencing, and *UBE3A* MLPA analysis (SALSA MLPA P336‐A2, MRC Holland, Amsterdam, The Netherlands). In addition, subtelomeric MLPA (SALSA MLPA P070), Autism MLPA (SALSA MLPA P343‐C1) and 60K array‐based comparative genomic hybridization (aCGH) were performed with normal results.

### 2.3 Whole‐exome sequencing

Trio whole‐exome sequencing of the patient and his parents was performed using the SureSelect Human All Exon V5+UTR kit (Agilent Technologies, Santa Clara, CA, USA). Sequencing was performed on an Illumina Hiseq2000 platform (Illumina, San Diego, CA, USA) producing 2x100nt paired‐end reads at the National Centre of Genomic Analysis (CNAG‐CRG, Barcelona, Spain). High‐quality reads were aligned to the GRCh37 decoy reference genome used by the 1000 genomes project (hs37d5) using the GEM3 aligner and variants identified following GATK Best Practices (DePristo et al., 2011). Coverage was assessed using GATK Depth of Coverage while ignoring reads with mapping quality <20 and bases with base quality <30.

All exome variants were filtered for allele frequencies <0.001 in the ExAC database (Lek et al., 2016), and their predicted impact on the protein (nonsense, frameshift, splice site, and missense variants were prioritized). The final candidate variant was confirmed by direct Sanger sequencing in the patient and excluded in his parents.

### 2.4 RNA analysis

RNA was extracted from isolated peripheral blood buffy coat stored at −196°C in liquid nitrogen using the Biostic Blood Total RNA Isolation Kit Sample (MO BIO Laboratories, Inc.), and cDNA synthesis was carried out using the PrimeScript^™^ RT reagent Kit (Takara Bio Inc.). Primers amplifying the region, including exons 3, 4, 5, 6, and 7 of *SMARCE1* gene (NCBI RefSeq NM_003079.4), were designed in order to analyze mRNA splicing.

## 3 RESULTS

Trio whole‐exome analysis identified a splice site variant c.237+1G>T in the *SMARCE1* gene (NCBI RefSeq NM_003079.4) after filtering the data according to a dominant *de novo* model of inheritance, a population allele frequency of <1/1,000 and a predicted impact on the protein. Variants in the *SMARCE1* gene are known to cause Coffin–Siris syndrome (CSS5; Coffin–Siris syndrome 5, OMIM 616938), which is a rare congenital syndrome affecting many organs, characterized by moderate to severe intellectual disability (Kosho & Okamoto, 2014; Santen, Emmelien, Vulto‐van Silfhout, Pottinger, & Van Bon, 2013).

**Figure 1:**
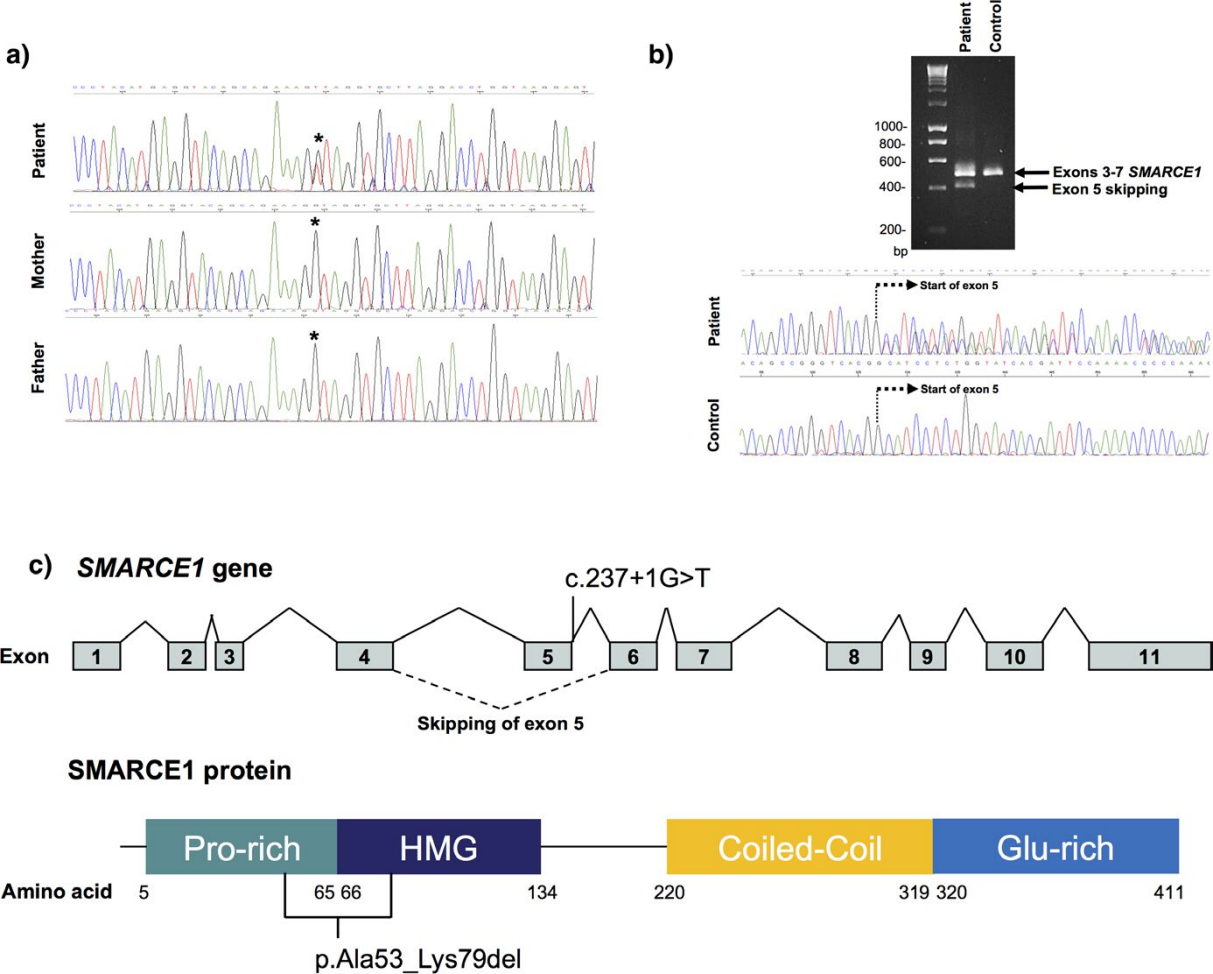
Molecular characterization of the *SMARCE1* (NCBI RefSeq NM_003079.4) c.237+1G>T splicing variant. (a) Sanger sequencing chromatographs showing the *SMARCE1* c.237+1G>T variant in the patient but not in his unaffected parents. The variant is indicated by black asterisks. (b) cDNA analysis of the *SMARCE1* c.237+1G>T variant in peripheral blood. PCR amplification products of exons 3 to 7 were run in a gel electrophoresis. The patient showed two bands compared to the negative control. Sanger sequencing chromatographs indicate skipping of exon 5. The start of the exon is indicated by a dashed line. (c) Schematic structure of the human *SMARCE1* gene and protein illustrating the predicted variant effect on splicing and protein. Exons are shown as boxes and introns as lines. Protein functional domains are shown as boxes. The amino acid deletion is delimited by black lines. Proline‐rich domain (Pro‐rich) 5–65 aa, High Mobility Group (HMG) 66–134 aa, Coiled‐Coil domain 220–319 aa, glutamic acid‐rich domain (Glu‐rich) 320–411 aa

The presence of the variant was confirmed by Sanger sequencing in the index patient, whereas the variant was not detected in his unaffected parents (Figure 1a). In order to analyze the effect of the splice site variant on mRNA processing, cDNA analysis was performed on the patient and a control sample. Amplification of exons 3 to 7 resulted in an additional smaller fragment in the patient suggesting exon skipping. Sanger sequencing of the cDNA confirmed the skipping of exon 5 in the patient sample (Figure 1b). Deletion of exon 5 results in an in‐frame deletion of 27 amino acids, removing the last part of the Pro‐rich (Proline‐rich) domain and the start of the HMG (High Mobility Group) domain (Figure 1c), which is essential for the proper functioning of the protein (Lomelí & Castillo‐Robles, 2016).

## 4 DISCUSSION

Using trio whole‐exome sequencing, we identified a novel splice site variant resulting in an in‐frame deletion in the *SMARCE1* gene in a patient with an AS‐like phenotype. Pathogenic variants in *SMARCE1* and another five genes (*SMARCB1, SMARCA4, SMARCA2, ARID1A,* and *ARID1B*) encoding subunits of the switch/sucrose non‐fermenting (SWI/SNF) ATP remodeling complex cause CSS (Miyake, Tsurusaki, & Matsumoto, 2014) which is a rare congenital syndrome characterized by developmental delay, moderate to severe intellectual disability, hypoplasic or absent fifth fingernails or toenails, distinctive facial features, hypertrichosis, sparse scalp hair, and hypotonia (Kosho & Okamoto, 2014; Santen et al., 2013; Tsurusaki et al., 2014; Zarate et al., 2016).

Germline *SMARCE1* heterozygous loss‐of‐function variants have been found in young patients with cranial and spinal meningiomas, consistent with a tumor suppressor mechanism (Lomelí & Castillo‐Robles, 2016; Smith et al., 2014) while missense variants cause Coffin–Siris syndrome (Kosho & Okamoto, 2014).

Here, we describe a splicing variant (c.237+1G>T) in the *SMARCE1* gene leading to an in‐frame deletion of 27 amino acids, removing part of the HMG domain (Figure 1c). The patient presents CSS clinical features. No spinal or intracranial meningiomas, which are characteristic of pathogenic loss‐of‐function variants in *SMARCE1*, were detected in a recent MRI, which showed a dysgenesis and hypoplasia of the corpus callosum and a global dilatation of the ventricular system, characteristic of Coffin–Siris patients. Interestingly, Smith et al., (2013) reported a splicing variant similar to the variant found in our patient, c.237+2T>C. It was identified in two members of a family with multiple spinal meningiomas with no clinical symptoms of CSS. RNA analysis of the affected individuals presented two alternatively spliced *SMARCE1* transcripts, one leading to a premature STOP codon and a less abundant second transcript leading to the same in‐frame deletion as that in our patient;. In our patient, the c.237+1G>T variant leads only to one alternative splice variant, the 27 amino acid in‐frame deletion, suggesting that the abundance of the in‐frame deletion transcript leads to a gain of function or dominant negative effect (Tsurusaki et al., 2012) and the appearance of a CSS phenotype.

The patient described here shows almost all the consistent and frequent clinical features associated to AS (Williams, Driscoll, & Dagli, 2010) except for the microcephaly and the frequent laughter/smiling (Table 1). Also, other associated AS features such as attraction to water, a happy demeanor, or frequent drooling are present. This led to the clinical diagnosis of AS which was not confirmed molecularly.

**Table 1:** Patient clinical features associated to AS and CSS

Clinical features associated to AS and CSS	Present in the patient
Development delay	✓
Severe mental retardation	✓
Speech impairment	✓
Receptive and non‐verbal communication skills higher than verbal ones	✓
Seizures	✓
Hypotonia	✓
Suck/swallowing disorders	✓
Hyperactivity	✓
Autistic features	✓
Strabismus	✓
Wide mouth	✓
Clinical features associated to AS^a^ but not to CSS	
Ataxia of gait	✓
Frequent laughter/smiling	‐
Apparent happy demeanor	✓
Easily excitable personality	✓
Attention deficit	✓
Hand‐flapping/stereotypies	✓
Microcephaly	‐
Abnormal EEG	✓ (not the characteristic of AS)
Clinical features associated to CSS^b^ but not to AS	
Small nails on 5th finger or toe	✓
Dysgenesis and hypoplasia of the corpus callosum	✓
Coarse facies	✓
Thick eyebrows	✓
Long eyelashes	✓
Broad nasal tip	✓
Thick vermilion of the lower lip	✓
Hypertrichosis	✓
Low anterior hairline	‐
Sparse scalp hair	✓
Joint laxity	✓

^a^Present in >80% of AS patients (Williams et al., 2010). ^b^Present in >60% of CSS patients (Schrier Vergano, Santen, Wieczorek, Wollnik, & Matsumoto, 2018).

**Figure 2:**
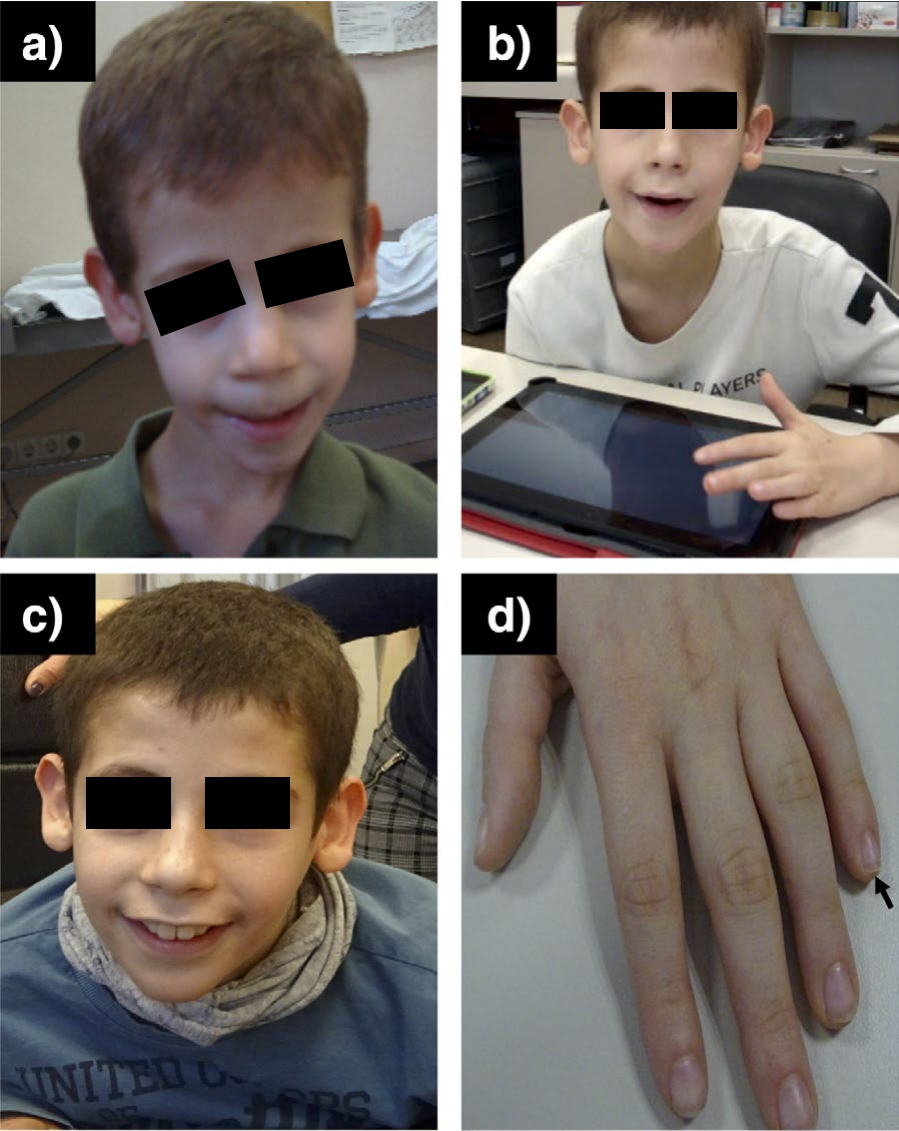
Patient clinical features. (a) Patient at 5 years of age, (b) 9 years of age, (c) 14 years of age, (d) Left hand showing small and hypoplastic 5th fingernail

Clinical reevaluation of the patient after the identification of the *SMARCE1* pathogenic variant showed the presence of clinical features associated to CSS but not to AS (Table 1). Among them, the characteristic hypoplasic nail on the 5th finger of the left hand, a coarse facies, sparse scalp hair, hypertricosis in the back and dysgenesis and hypoplasia of the corpus callosum (Figure 2). CSS patients, like AS patients, present severe developmental delay, speech impairment with expressive language more severely affected than receptive language, moderate to severe intellectual disability and behavioral abnormalities such as hyperactivity and autistic features (Table 1) reflecting the clinical overlap between the two syndromes. However, the patient described here presents some other features characteristic of AS which have not been described in CSS before such as the ataxia of gait and the stereotypes.

To date, only six individuals with *SMARCE1* missense pathogenic variants have been reported (Zarate et al., 2016). Here, we describe the first patient with a pathogenic splicing variant in the CSS gene, *SMARCE1*, who had a diagnosis of AS‐like and who presents some clinical features characteristic of AS, which have not been previously associated to CSS. Taking into account these results, we believe that CSS should be added to the expanding list of differential diagnoses for AS, probably accounting for some of the molecularly undiagnosed AS‐like patients.

The increasing use of exome sequencing in diagnostic laboratories will allow the analysis of all those genes that are involved in severe neurodevelopment disorders in patients who present AS clinical features, improving the diagnostic rate, and providing knowledge of the phenotypic spectrum of AS‐like causative genes, among them those responsible for Coffin–Siris syndrome.

## CONFLICT OF INTEREST

The authors declare no conflict of interest.

## References

[mgg3511-bib-0001] Berger, S. I. , Ciccone, C. , Simon, K. L. , Malicdan, M. C. , Vilboux, T. , Billington, C. , … Smith, A. C. M. (2017). Exome analysis of Smith–Magenis‐like syndrome cohort identifies de novo likely pathogenic variants. Human Genetics, 136, 409–420. 10.1007/s00439-017-1767-x 28213671 PMC5848494

[mgg3511-bib-0002] Bird, L. (2014). Angelman syndrome: review of clinical and molecular aspects. The Application of Clinical Genetics, 7, 93–104. 10.2147/TACG 24876791 PMC4036146

[mgg3511-bib-0003] Buiting, K. (2010). Prader‐Willi syndrome and Angelman syndrome. American Journal of Medical Genetics Part C, 154C, 365–376. 10.1002/ajmg.c.30273 20803659

[mgg3511-bib-0004] Dagli, A. , Buiting, K. , & Williams, C. A. (2012). Molecular and clinical aspects of Angelman syndrome. Molecular Syndromology, 2, 100–112. 22670133 10.1159/000328837 PMC3366701

[mgg3511-bib-0005] DePristo, M. A. , Banks, E. , Poplin, R. , Garimella, K. V. , Maguire, J. R. , Hartl, C. , … Daly, M. J. (2011). A framework for variation discovery and genotyping using next‐generation DNA sequencing data. Nature Genetics, 43, 491–498. 10.1038/ng.806 21478889 PMC3083463

[mgg3511-bib-0006] Kleefstra, T. , Kramer Jamie, M. , Neveling, K. , Willemsen Marjolein, H. , Koemans Tom, S. , Vissers Lisenka, E. L. M. , … van Bokhoven, H. (2012). Disruption of an EHMT1‐associated chromatin‐modification module causes intellectual disability. American Journal of Human Genetics, 91, 73–82. 10.1016/j.ajhg.2012.05.003 22726846 PMC3397275

[mgg3511-bib-0007] Kosho, T. , & Okamoto, N. (2014). Genotype‐phenotype correlation of Coffin‐Siris syndrome caused by mutations in SMARCB1, SMARCA4, SMARCE1, and ARID1A. American Journal of Medical Genetics Part C, 166, 262–275. 10.1002/ajmg.c.31407 25168959

[mgg3511-bib-0008] Lek, M. , Karczewski, K. J. , Minikel, E. V. , Samocha, K. E. , Banks, E. , Fennell, T. , … MacArthur, D. G. (2016). Analysis of protein‐coding genetic variation in 60,706 humans. Nature, 536, 285–291. 10.1038/nature19057 27535533 PMC5018207

[mgg3511-bib-0009] Lomelí, H. , & Castillo‐Robles, J. (2016). The developmental and pathogenic roles of BAF57, a special subunit of the BAF chromatin‐remodeling complex. FEBS Letters, 590, 1555–1569. 10.1002/1873-3468.12201 27149204

[mgg3511-bib-0010] Miyake, N. , Tsurusaki, Y. , & Matsumoto, N. (2014). Numerous BAF complex genes are mutated in Coffin‐Siris syndrome. American Journal of Medical Genetics Part C, 166, 257–261. 10.1002/ajmg.c.31406 25081545

[mgg3511-bib-0011] Sajan, S. A. , Jhangiani, S. N. , Muzny, D. M. , Gibbs, R. A. , Lupski, J. R. , Glaze, D. G. , … Neul, J. L. (2016). Enrichment of mutations in chromatin regulators in people with Rett syndrome lacking mutations in MECP2. Genetics in Medicine, 19, 13–19. 27171548 10.1038/gim.2016.42 PMC5107176

[mgg3511-bib-0012] Santen, G. W. E. , Emmelien, A. , Vulto‐van Silfhout, A. T. , Pottinger, C. , & Van Bon, B. W. M. (2013). Coffin‐Siris syndrome and the BAF complex: Genotype‐phenotype study in 63 patients. Human Mutation, 34, 1519–1528. 10.1002/humu.22394 23929686

[mgg3511-bib-0013] Schrier Vergano, S. , Santen, G. , Wieczorek, D. , Wollnik, B. , & Matsumoto, N. (2018). Coffin‐siris syndrome In Adam M. P., Ardinger H. H., Pagon R. A., Wallace S. E., Bean L. J. H., Stephens K., & Amemiya A. (Eds.), GeneReviews® [Internet]. (pp. 1993‐2018). Seattle, WA: University of Washington. 23556151

[mgg3511-bib-0014] Smith, M. J. , O'Sullivan, J. , Bhaskar, S. S. , Hadfield, K. D. , Poke, G. , Caird, J. , … Evans, D. G. (2013). Loss‐of‐function mutations in SMARCE1 cause an inherited disorder of multiple spinal meningiomas. Nature Genetics, 45, 295–298. 10.1038/ng.2552 23377182

[mgg3511-bib-0015] Smith, M. J. , Wallace, A. J. , Bennett, C. , Hasselblatt, M. , Elert‐Dobkowska, E. , Evans, L. T. , … Evans, D. G. (2014). Germline SMARCE1 mutations predispose to both spinal and cranial clear cell meningiomas. The Journal of Pathology, 234, 436–440. 10.1002/path.4427 25143307

[mgg3511-bib-0016] Tan, W.‐H. , Bird, L. M. , Thibert, R. L. , & Williams, C. A. (2014). If not Angelman, what is it? A review of Angelman‐like syndromes. American Journal of Medical Genetics. Part A, 164, 975–992. 10.1002/ajmg.a.36416 24779060

[mgg3511-bib-0017] Tsurusaki, Y. , Okamoto, N. , Ohashi, H. , Kosho, T. , Imai, Y. , Hibi‐Ko, Y. , … Matsumoto, N. (2012). Mutations affecting components of the SWI/SNF complex cause Coffin‐Siris syndrome. Nature Genetics, 44, 376 10.1038/ng.2219 22426308

[mgg3511-bib-0018] Tsurusaki, Y. , Okamoto, N. , Ohashi, H. , Mizuno, S. , Matsumoto, N. , Makita, Y. , … Miyake, N. (2014). Coffin‐Siris syndrome is a SWI/SNF complex disorder. Clinical Genetics, 85, 548–554. 10.1111/cge.12225 23815551

[mgg3511-bib-0019] Williams, C. A. , Driscoll, D. J. , & Dagli, A. I. (2010). Clinical and genetic aspects of Angelman syndrome. Genetics in Medicine, 12, 385–395. 20445456 10.1097/GIM.0b013e3181def138

[mgg3511-bib-0020] Zarate, Y. A. , Bhoj, E. , Kaylor, J. , Li, D. , Tsurusaki, Y. , Miyake, N. , … Schrier Vergano, S. A. (2016). SMARCE1, a rare cause of Coffin‐Siris syndrome: Clinical description of three additional cases. American Journal of Medical Genetics. Part A, 170, 1967–1973. 10.1002/ajmg.a.37722 27264197 PMC5870868

